# Triple-high expression of phosphatase and tensin homolog (PTEN), estrogen receptor (ER) and progesterone receptor (PR) may predict favorable prognosis for patients with Type I endometrial carcinoma

**DOI:** 10.7150/jca.33720

**Published:** 2020-01-13

**Authors:** Yanfang Liang, Bihua Lin, Ziyu Ye, Shasha Chen, Haibo Yu, Can Chen, Xin Zhang, Keyuan Zhou, Jincheng Zeng

**Affiliations:** 1 Department of Pathology, Dongguan Hospital Affiliated to Medical College of Jinan University, The Fifth People's Hospital of Dongguan, Dongguan 523905, China; 2 Dongguan Key Laboratory of Medical Bioactive Molecular Developmental and Translational Research, Guangdong Provincial Key Laboratory of Medical Molecular Diagnostics, Guangdong Medical University, Dongguan, Guangdong 523808, China; 3 Clinical Experimental Center, Jiangmen Central Hospital, Affiliated Jiangmen Hospital of Sun Yat-sen University, Jiangmen 529030, China.

**Keywords:** Endometrial carcinoma, estrogen receptors, progesterone receptor, phosphatase and tensin homolog, prognosis

## Abstract

Endometrial carcinoma (EC) is the most common malignant tumors in female derived from the endometrial epithelium. Several previous studies have described estrogen receptors (ER), progesterone Receptor (PR) and phosphatase and tensin homolog (PTEN) are associated with clinicopathological factors and prognosis in EC patients. However, during EC patients follow-up, we found that some EC patients with down-regulation of PTEN, but up-regulation of ER or PR , and some EC patients with down-regulation of ER or PR, but up-regulation of PTEN also had a poor prognosis. Therefore, to reveal the prognosis of EC patients with different phenotypes based on PTEN, ER and PR expression, 120 cases formalin-fixed paraffin-embedded EC tissues and 543 cases uterine corpus endometrial carcinoma (UCEC) patients from the cancer genome atlas (TCGA) UCEC datasets were analyzed. Results showed that EC tissues can be classified to PTEN^L^ER^L^PR^L^, PTEN^H^ER^L^PR^L^, PTEN^H^ER^H^PR^H^, PTEN^L^ER^H^PR^H^, PTEN^H^ER^H^PR^L^, PTEN^H^ER^L^PR^H^, and PTEN^L^ER^H^PR^L^ phenotypes basing on IHC analysis. Additionally, EC patients with PTEN^L^ER^L^PR^L^ showed high malignancy, while patients with PTEN^H^ER^H^PR^H^ showed low malignancy. Therefore, combined detection of PTEN, ER, PR may help identify a small subset of EC with more aggressive behavior and may aid in risk stratification.

## Introduction

Endometrial carcinoma (EC) is the most common malignant tumors in female derived from the endometrial epithelium. Recently, the EC incidence is increased, while the 5-year survival rate is decreased 1. The etiology of EC is not yet clear. Experimental and epidemiologic evidence showed that the risk factors of EC including BMI≥25kg/m^2^, hypertension, diabetes, smoking, taking tamoxifen, family history of cancer and non-pregnant women^2^, ^3^. EC can be broadly classified into two types. Majority (~80%) of EC patients are of Type I endometrioid histology, up to 15% are Type II EC patients, primarily serous carcinomas 3-5. Most of Type I EC patients present with low-grade and early-stage disease and have a favorable prognosis. According to the previous study, 74-91% of International Federation of Gynecology and Obstetrics (FIGO) stages I-II type I EC patients have a 5-year OS time 6. However, 10-20% of early stages I-II and 50-70% of advanced stage III-IV type I EC patients will recur after primary treatment 7. Previous studies have revealed that clinicopathological parameters such as histological grade, FIGO clinical stage, myometrial tumor invasion, tumor size, lymph node metastasis, lymphovascular space invasion, and the patients' age and race has prognostic effect in type I EC patients^6^, ^8^, ^9^. However, these factors are usually obtained postoperatively and have proven to be insufficient to predict recurrence and estimate survival time. Therefore, it is necessary to identify more effective prognostic predictors to identify preoperative high-risk type I EC patients.

Several previous studies have described estrogen receptors (ER), progesterone Receptor (PR) and phosphatase and tensin homolog (PTEN) are associated with clinicopathological factors and prognosis in EC patients 10-16. Both of ER and PR are members of the nuclear receptor family that release relevant related ligand-activated transcription factors to regulate cell gene expression through activation and suppression of transcription 17-19. Low expression or deletion of ER and PR associated with increased malignancy, invasion, and non-hormone-dependent tumor transformation 20. The absence of PR and ER indicates poor prognosis, poor histologic type, higher histological grade, higher frequency of lymph node metastasis, and higher clinical staging at diagnosis 18, 19. PTEN located on chromosome 10q23.3, is known as a vital tumor suppressor gene. Recently, researchers found that PTEN has low expression during the development and progression of EC, and implies poor prognosis, higher histological grade and clinical staging, and shorter survival 21, 22. Especially, the mutation rate of *PTEN* in EC is about 34% ~ 55%, which is higher than the mutation rate of *K-ras* and *P53* 23, 24. However, during follow-up of EC patients, we found that some EC patients with down-regulation of PTEN, but up-regulation of ER or PR, and some EC patients with down-regulation of ER or PR, but up-regulation of PTEN also had a poor prognosis. Therefore, to reveal the prognosis of EC patients with different phenotypes based on PTEN, ER and PR expression, 120 cases formalin-fixed paraffin-embedded EC tissues and 543 cases UCEC patients from TCGA-UCEC datasets were analyzed.

## Material and Methods

### Patients

A total of 120 formalin-fixed paraffin embedded EC tissue samples between March 2005 and April 2015 from Affiliated Hospital of Guangdong Medical University (China), Dongguan Fifth People's Hospital (China), and Affiliated Jiangmen Hospital of Sun Yat-sen University (China) were collected. Tumors were staged according to the FIGO 2009 system 25. Histological grade was assessed based on the 2014 World Health Organization criteria 26. Patients, who had history of other tumors, underwent chemotherapy, radiotherapy, radical surgery treatment, or other anticancer therapies prior to surgery were excluded in this study. The demographic and clinical characteristics for all the patients are shown in Table 1. Informed consent was obtained from all study subjects, and the studies were approved by the institutional ethics committee.

### Immunohistochemistry (IHC)

Tissue sections (4-μM) were prepared from formalin-fixed paraffin embedded tissue blocks and then subjected to stain with hematoxylin and eosin as our previously described 27-29. The results were assessed by two pathologists to demonstrate the presence of tumor and the proportion of tumor cells in each section. Tissue sections were subjected to incubate in 0.3% Hydrogen peroxide solution for 10 min at room temperature to block the endogenous peroxidase activity, and then washed by Phosphate Buffered Saline (PBS) solution. Antigenic epitopes were next retrieved by heating for 2 min in 10 mmol/L citrate buffer (pH 6.0). The slides were then first incubated with antibodies against PTEN (ZSGB-BIO, China), ER (ZSGB-BIO, China), PR (ZSGB-BIO, China), Ki-67 (Thermofisher,USA), p53 (Thermofisher,USA), CEA (Thermofisher,USA) and CA125 (Thermofisher,USA) for 30 min at room temperature. Next, the sections were washed with PBS for three times and followed by a goat anti-rabbit and mouse IgG-HRP (Kit-0015, Maixin Biotech, Fuzhou, China) secondary antibody for one hour at room temperature at 1:500 dilutions. The slides were visualized using DAB Detection Kit (Enhanced Polymer) (Kit-0015, Maixin Biotech, Fuzhou, China) and chromogenic reaction was controlled under a microscope (Nikon). After immunostaining, sections were immersed into hematoxylin for nuclear staining, then dehydrated through gradient concentrations of ethanol, cleared with xylene, and covered with neutral balsam.

### Score of immunohistochemical sections

The score of immunohistochemical sections were assessed by two pathologists in a blinded fashion to the clinical status of the patients, as our previously reported 27-29. The immunoreactive area (percentage of positive staining cells) and intensity scores of PTEN, ER and PR were evaluated. In brief, according to the immunoreactive area, 0-5% scored 0, 6-25% scored 1, 26-50% scored 2, 51~75% scored 3 and more than 75% scored 4. If the scores was 0~2, the section was defined as low expression, and if the final scores was 3~6 was defined as high expression. Two specialists who were blinded to the clinical status of the patients evaluated the staining independently.

### mRNA expression

All available mRNA expression data were collected from 543 UCEC tumors and 23 adjacent non-EC tissues in TCGA (https://cancergenome.nih.gov/). According to the average value of mRNA expression of each gene, EC patients were divided into 8 phenotypes (*PTEN*^L^*ESR*1^L^*PGR*^L^, *PTEN*^L^*ESR1*^H^*PGR*^H^, *PTEN*^H^*ESR1*^L^*PGR*^L^, *PTEN*^H^*ESR1*^H^*PGR*^H^, *PTEN*^L^*ESR1*^L^*PGR*^H^, *PTEN*^L^*ESR1*^H^*PGR*^L^, *PTEN*^H^*ESR1*^H^*PGR*^L^, and *PTEN*^H^*ESR1*^L^*PGR*^H^), according to high and low *PTEN*,* PGR* and* ESR1* mRNA expression.

### Statistical analysis

Statistical analysis was performed using SPSS 19.0 Software (SPSS, Chicago, IL, USA). Chi-square test or Fisher's exact test was employed for analysis the differences of categorical variables. For survival analysis, overall survival (OS) or disease-free survival (DFS) was calculated using Kaplan-Meier method and evaluated by log-rank test, as our previously reported 30, 31. Multivariate analysis was based on the Cox proportional hazard regression model. A p value <0.05 was considered with statistical significance.

## Results

### Classification of EC tissues based on PTEN, ER and PR expression

**Figure 1:**
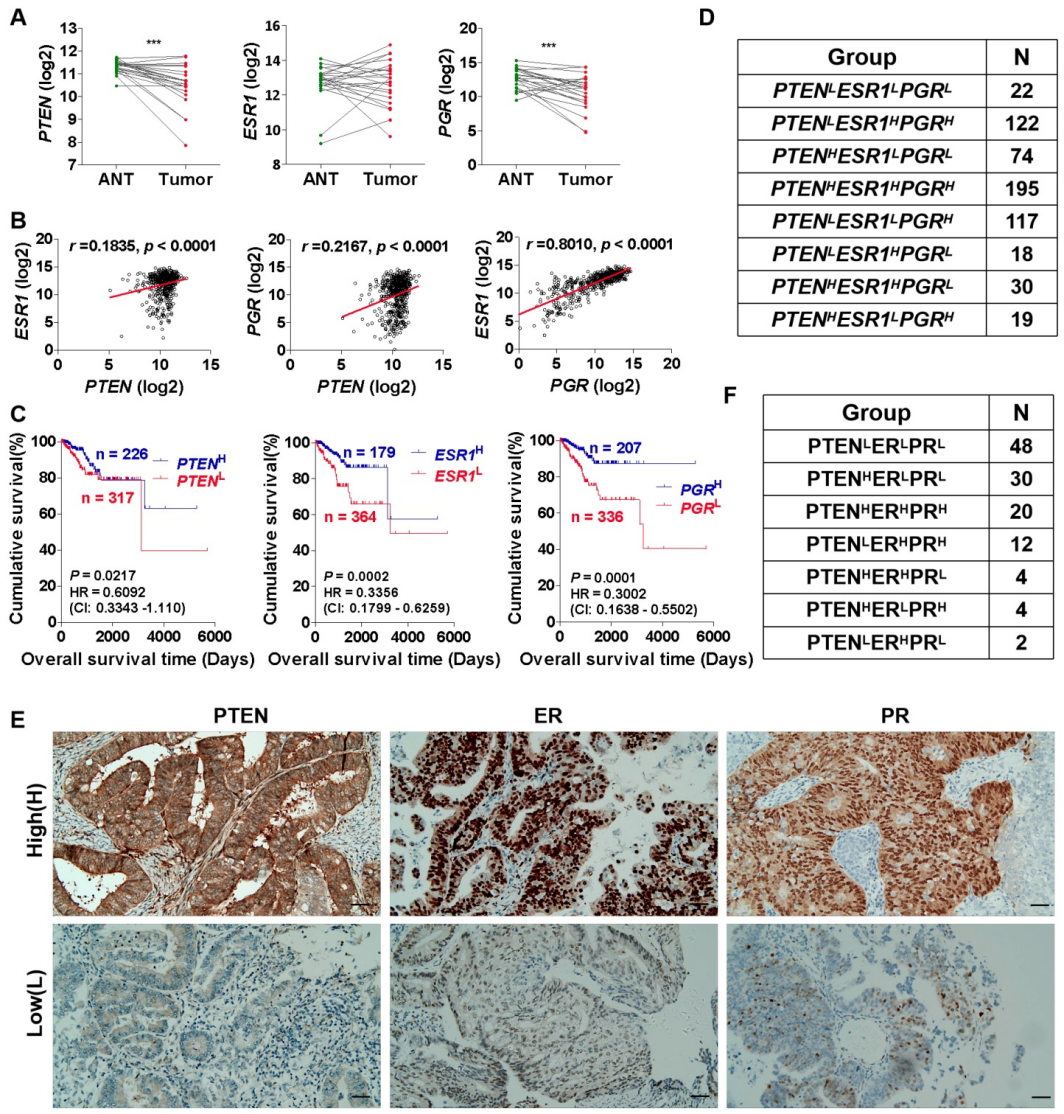
** Classification of EC tissues based on PTEN, ER and PR expression. (A)** Comparison of *PTEN*, *PGR* and *ESR1* mRNA expression between EC tumor tissues and adjacent normal tissues (ANT) based on TCGA database, respectively. ***: *p* < 0.001. **(B)** Correlation analysis among *PTEN*, *PGR* and *ESR1* mRNA expression. **(C)** Kaplan-Meier analysis of overall survival time of EC patients with high *PTEN* mRNA expression versus low *PTEN* mRNA expression, high* PGR* mRNA expression versus low *PGR* mRNA expression and high *ESR1* mRNA expression versus low *ESR1* mRNA expression, respectively, based on TCGA database. **(D)** EC patients were divided into 8 phenotypes according to high and low *PTEN*,* PGR* and* ESR1* mRNA expression based on TCGA database. **(E)** Detection of PTEN, ER and PR expression in EC tissues by IHC analysis. **(F)** EC patients were divided into 7 phenotypes according to high and low PTEN, ER and PR expression based on IHC analysis. H: high, L: low.

It has been reported that the tumor suppressor gene PTEN is down-regulated in a variety of cancers, including breast cancer 32, prostate cancer 32 and EC 33, etc. PTEN deficiency accelerates tumuor progression and invasiveness 34, promotes macrophage infiltration 35, and plays a significant role in the pathogenesis of carcinogenesis 36. Herein, we first analyzed the cancer genome atlas uterine corpus endometrial carcinoma (TCGA-UCEC) datasets and found that *PTEN* mRNA expression was down-regulated in EC tumor tissues compared with adjacent normal tissues (ANT) (Fig. 1A). Prognostic factors of EC include the presence of ER and PR. We also found that the mRNA expression of *PGR* encoding PR, but not *ESR1* encoding ER, down-regulated in EC tissues compared with ANT in TCGA-UCEC datasets (Fig. 1A). Furthermore, correlation analysis showed that there was a significant correlation among *PTEN*,* PGR* and* ESR1* mRNA expression (Fig. 1B), and they all associated with the prognosis of EC (Fig. 1C). This was also consistent with the results reported in most previous studies 18, 19, 21, 22. To further reveal the relationship between differential expression of PTEN, ER and PR, and EC prognosis, EC patients were divided into 8 phenotypes (*PTEN*^L^*ESR*1^L^*PGR*^L^, *PTEN*^L^*ESR1*^H^*PGR*^H^, *PTEN*^H^*ESR1*^L^*PGR*^L^, *PTEN*^H^*ESR1*^H^*PGR*^H^, *PTEN*^L^*ESR1*^L^*PGR*^H^, *PTEN*^L^*ESR1*^H^*PGR*^L^, *PTEN*^H^*ESR1*^H^*PGR*^L^, and *PTEN*^H^*ESR1*^L^*PGR*^H^), according to high (H) and low (L) *PTEN*,* PGR* and* ESR1* mRNA expression (Fig. 1D). Additionally, we collected 120 formalin-fixed paraffin-embedded EC tissues and examined PTEN, ER and PR expression by IHC analysis (Fig. E). Based on PTEN, ER and PR expression, EC tissues can be classified to PTEN^L^ER^L^PR^L^ (48/120), PTEN^H^ER^L^PR^L^ (30/120), PTEN^H^ER^H^PR^H^ (20/120), PTEN^L^ER^H^PR^H^ (12/120), PTEN^H^ER^H^PR^L^ (4/120), PTEN^H^ER^L^PR^H^ (4/120), and PTEN^L^ER^H^PR^L^ (2/120) phenotype (Fig. 1F). The demographic and clinical characteristics for all EC phenotypes are shown in Table 1. 60% of EC patients with PTEN^H^ER^L^PR^L^ and PTEN^H^ER^H^PR^H^ phenotype were G1 histological grading, respectively, while 20.83% of EC patients with PTEN^L^ER^L^PR^L^ phenotype were G1 histological grading (Table 1). Similarly, in FIGO clinical staging, most EC patients with PTEN^H^ER^L^PR^L^ (66.7%) and PTEN^H^ER^H^PR^H^ (45.0%) phenotype were stage I, while 25.00% of EC patients with PTEN^L^ER^L^PR^L^ phenotype were stage I (Table 1). These results suggest that different EC phenotypes which classified by PTEN, ER and PR expression may be associated with clinical pathological and histological grading.

### EC patients with triple-high expression of PTEN, ER and PR showed a lower degree of malignancy and proliferative activity

**Figure 2:**
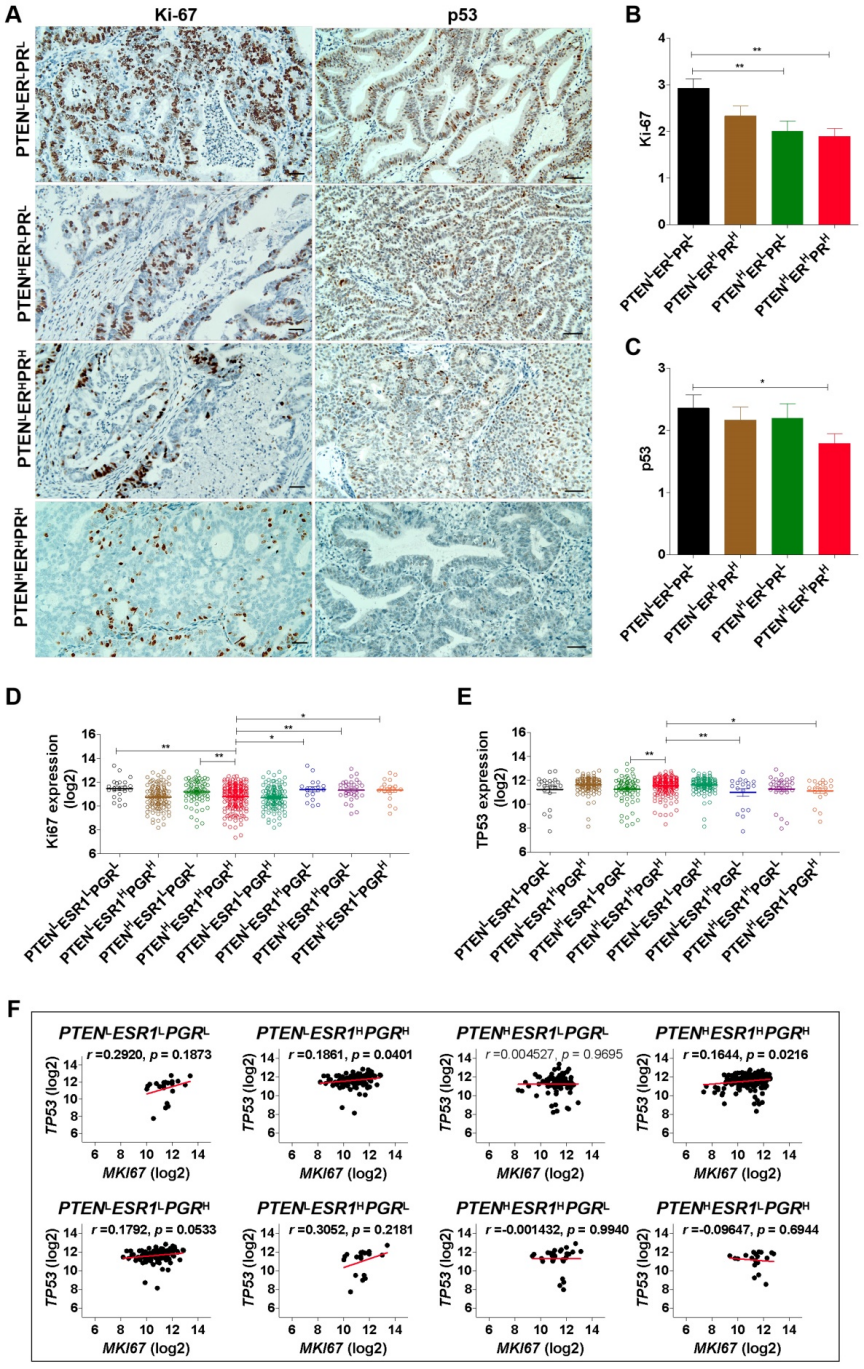
** EC patients with triple-H expression of PTEN, ER and PR showed a Ler expression of Ki-67 and p53. (A)** Detection of Ki-67 and p53 expression in EC tissues with PTEN^L^ER^L^PR^L^, PTEN^H^ER^L^PR^L^, PTEN^H^ER^H^PR^H^ and PTEN^L^ER^H^PR^H^ phenotypes by IHC analysis. **(B)** Comparison of Ki-67 expression among PTEN^L^ER^L^PR^L^, PTEN^H^ER^L^PR^L^, PTEN^H^ER^H^PR^H^ and PTEN^L^ER^H^PR^H^ phenotypes. **:* p* < 0.01. **(C)** Comparison of *Ki67* mRNA expression among *PTEN*^L^*ESR*1^L^*PGR*^L^, *PTEN*^L^*ESR1*^H^*PGR*^H^, *PTEN*^H^*ESR1*^L^*PGR*^L^, *PTEN*^H^*ESR1*^H^*PGR*^H^, *PTEN*^L^*ESR1*^L^*PGR*^H^, *PTEN*^L^*ESR1*^H^*PGR*^L^, *PTEN*^H^*ESR1*^H^*PGR*^L^, and *PTEN*^H^*ESR1*^L^*PGR*^H^ phenotypes based on TCGA database. *: *p* < 0.05; **:* p* < 0.01. **(D)** Comparison of p53 expression among PTEN^L^ER^L^PR^L^, PTEN^H^ER^L^PR^L^, PTEN^H^ER^H^PR^H^ and PTEN^L^ER^H^PR^H^ phenotypes. **:* p* < 0.01. **(E)** Comparison of *TP53* mRNA expression among *PTEN*^L^*ESR*1^L^*PGR*^L^, *PTEN*^L^*ESR1*^H^*PGR*^H^, *PTEN*^H^*ESR1*^L^*PGR*^L^, *PTEN*^H^*ESR1*^H^*PGR*^H^, *PTEN*^L^*ESR1*^L^*PGR*^H^, *PTEN*^L^*ESR1*^H^*PGR*^L^, *PTEN*^H^*ESR1*^H^*PGR*^L^, and *PTEN*^H^*ESR1*^L^*PGR*^H^ phenotypes based on TCGA database. *: *p* < 0.05; **: *p* < 0.01.** (F)** Correlation analysis between *Ki67* and *TP53* mRNA expression in *PTEN*^L^*ESR*1^L^*PGR*^L^, *PTEN*^L^*ESR1*^H^*PGR*^H^, *PTEN*^H^*ESR1*^L^*PGR*^L^, *PTEN*^H^*ESR1*^H^*PGR*^H^, *PTEN*^L^*ESR1*^L^*PGR*^H^, *PTEN*^L^*ESR1*^H^*PGR*^L^, *PTEN*^H^*ESR1*^H^*PGR*^L^, and *PTEN*^H^*ESR1*^L^*PGR*^H^ phenotypes based on TCGA database. H: high, L: low.

To reveal the proliferative activity of EC patients with different phenotypes, Ki-67 and p53 were detected by IHC analysis (Fig. 2A). Results showed that Ki-67 was low expressed in EC patients with PTEN^H^ER^L^PR^L^ and PTEN^H^ER^H^PR^H^ phenotype, while high expressed in EC patients with PTEN^L^ER^L^PR^L^ phenotype (Fig. 2B). Indeed, based on TCGA-UCEC datasets, we also found that EC patients with *PTEN*^L^*ESR*1^L^*PGR*^L^ phenotype had high expression of *Ki67* mRNA, while patients with *PTEN*^H^*ESR1*^H^*PGR*^H^ phenotype had low expression of *Ki67* mRNA (Fig. 2C). Simultaneously, we also found p53 was low expressed in EC patients with PTEN^H^ER^H^PR^H^ phenotype, and *TP53* (encoding p53) mRNA was low expressed in EC patients with *PTEN*^H^*ESR1*^H^*PGR*^H^ phenotype (Fig. 2D, 2E). Interestingly, there was a positive correlation between *Ki67* and *TP53* mRNA expression in patients with *PTEN*^H^*ESR1*^H^*PGR*^H^ phenotype (*r* =0.1644; *p* = 0.0216) and *PTEN*^L^*ESR1*^H^*PGR*^H^ phenotype (*r* =0.1861; *p* = 0.0401), respectively, but not in *PTEN*^L^*ESR*1^L^*PGR*^L^, *PTEN*^H^*ESR1*^L^*PGR*^L^, *PTEN*^L^*ESR1*^L^*PGR*^H^, *PTEN*^L^*ESR1*^H^*PGR*^L^, *PTEN*^H^*ESR1*^H^*PGR*^L^, and *PTEN*^H^*ESR1*^L^*PGR*^H^ phenotype (Fig. 2F). These results suggest that EC patients with triple-high expression of PTEN, ER and PR showed a lower degree of malignancy and proliferative activity.

### EC patients with triple-high expression of PTEN, ER and PR showed a lower expression of CA125

**Figure 3:**
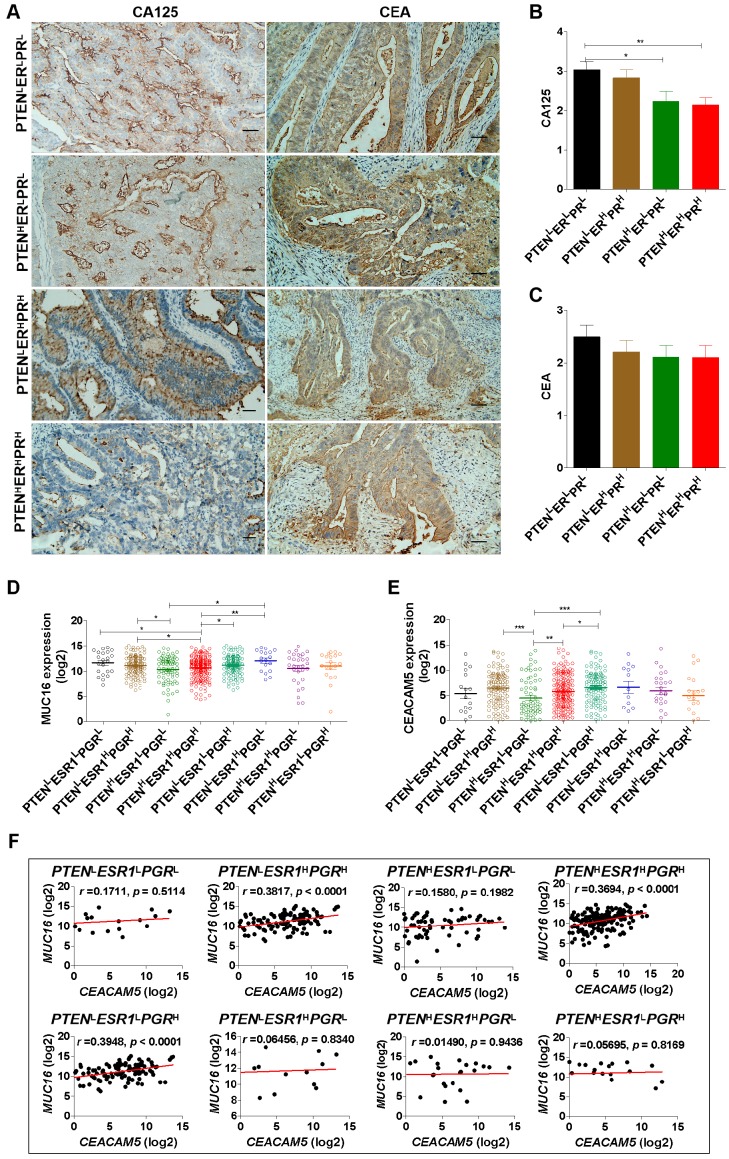
** EC patients with triple-H expression of PTEN, ER and PR showed a Ler expression of CA125. (A)** Detection of CA125 and CEA expression in EC tissues with PTEN^L^ER^L^PR^L^, PTEN^H^ER^L^PR^L^, PTEN^H^ER^H^PR^H^ and PTEN^L^ER^H^PR^H^ phenotypes by IHC analysis. **(B)** Comparison of CA125 expression among PTEN^L^ER^L^PR^L^, PTEN^H^ER^L^PR^L^, PTEN^H^ER^H^PR^H^ and PTEN^L^ER^H^PR^H^ phenotypes. **:* p* < 0.01. **(C)** Comparison of *MUC16* mRNA expression among *PTEN*^L^*ESR*1^L^*PGR*^L^, *PTEN*^L^*ESR1*^H^*PGR*^H^, *PTEN*^H^*ESR1*^L^*PGR*^L^, *PTEN*^H^*ESR1*^H^*PGR*^H^, *PTEN*^L^*ESR1*^L^*PGR*^H^, *PTEN*^L^*ESR1*^H^*PGR*^L^, *PTEN*^H^*ESR1*^H^*PGR*^L^, and *PTEN*^H^*ESR1*^L^*PGR*^H^ phenotypes based on TCGA database. *: *p* < 0.05; **: *p* < 0.01. **(D)** Comparison of CEA expression among PTEN^L^ER^L^PR^L^, PTEN^H^ER^L^PR^L^, PTEN^H^ER^H^PR^H^ and PTEN^L^ER^H^PR^H^ phenotypes. **:* p* < 0.01. **(E)** Comparison of *CEACAM5* mRNA expression among *PTEN*^L^*ESR*1^L^*PGR*^L^, *PTEN*^L^*ESR1*^H^*PGR*^H^, *PTEN*^H^*ESR1*^L^*PGR*^L^, *PTEN*^H^*ESR1*^H^*PGR*^H^, *PTEN*^L^*ESR1*^L^*PGR*^H^, *PTEN*^L^*ESR1*^H^*PGR*^L^, *PTEN*^H^*ESR1*^H^*PGR*^L^, and *PTEN*^H^*ESR1*^L^*PGR*^H^ phenotypes based on TCGA database. *: *p* < 0.05; **:* p* < 0.01.** (F)** Correlation analysis between *MUC16* and *CEACAM5* mRNA expression in *PTEN*^L^*ESR*1^L^*PGR*^L^, *PTEN*^L^*ESR1*^H^*PGR*^H^, *PTEN*^H^*ESR1*^L^*PGR*^L^, *PTEN*^H^*ESR1*^H^*PGR*^H^, *PTEN*^L^*ESR1*^L^*PGR*^H^, *PTEN*^L^*ESR1*^H^*PGR*^L^, *PTEN*^H^*ESR1*^H^*PGR*^L^, and *PTEN*^H^*ESR1*^L^*PGR*^H^ phenotypes based on TCGA database. H: high, L: low.

Carcinoma-associated antigens are considered to be useful markers for the detection of recurrent disease in EC patients 37. Herein, we detected CA125 and CEA expression in EC tissues by IHC analysis (Fig. 3A). The results show that EC patients with PTEN^H^ER^H^PR^H^ phenotype had low expression of CA125 (Fig. 3B) and EC patients with *PTEN*^H^*ESR1*^H^*PGR*^H^ phenotype had low expression of *MUC16* (encoding CA125) mRNA (Fig. 3C). Notably, there was no difference in the expression of CEA among patients with PTEN^L^ER^L^PR^L^, PTEN^H^ER^L^PR^L^, PTEN^H^ER^H^PR^H^, and PTEN^L^ER^H^PR^H^ phenotypes (Fig. 3D). However, the mRNA expression of *CEACAM5* (encoding CEA) was down-regulated in patients with* PTEN*^H^*ESR1*^H^*PGR*^H^ and *PTEN*^H^*ESR1*^L^*PGR*^L^ phenotype compared to patients with *PTEN*^L^*ESR1*^L^*PGR*^H^ phenotype based on TCGA-UCEC datasets (Fig. 3E). Furthermore, we observed *MUC16* mRNA expression was positively related to *CEACAM5* mRNA expression in EC patients with* PTEN*^H^*ESR1*^H^*PGR*^H^ (*r*=0.3694; *p* < 0.0001)*, PTEN*^L^*ESR1*^H^*PGR*^H^ (*r*=0.3817; *p* < 0.0001) and *PTEN*^L^*ESR1*^L^*PGR*^H^ (*r* =0.3948; *p* < 0.0001) phenotypes, respectively (Fig. 3F). These studies suggest that there are differences in CEA and CA125 expression in EC patients with different phenotypes. In particular, EC patients with triple-high expression of PTEN, ER and PR showed low expression of CA125, this was positively correlated with CEA.

### Triple-high expression of PTEN, ER and PR may predict favorable prognosis in EC patients

**Figure 4:**
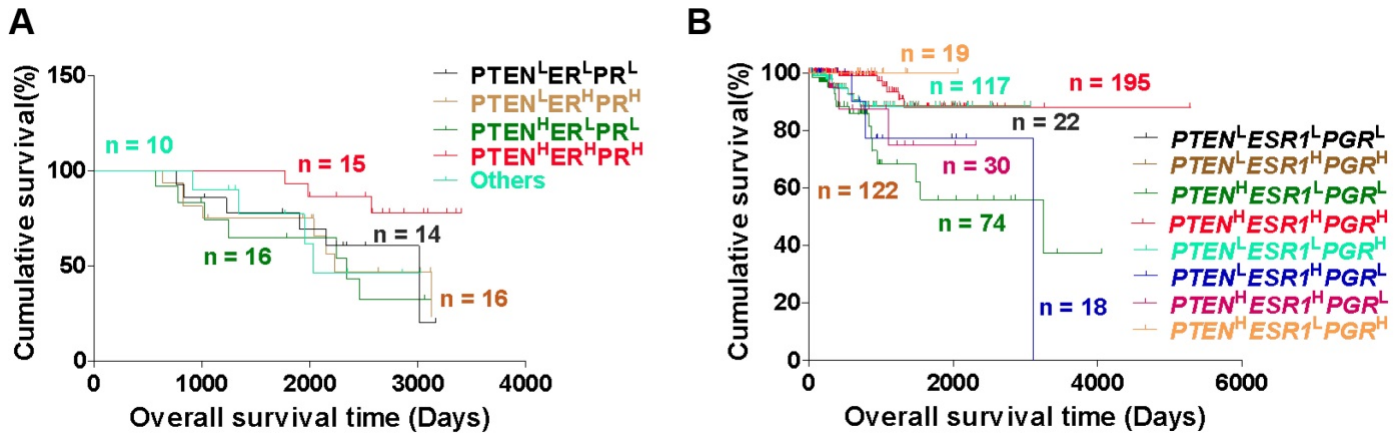
** Triple-H expression of PTEN, ER and PR may predict favorable prognosis in EC patients. (A)** Kaplan-Meier analysis of overall survival time of EC patients with PTEN^L^ER^L^PR^L^, PTEN^H^ER^L^PR^L^, PTEN^H^ER^H^PR^H^ and PTEN^L^ER^H^PR^H^ phenotypes. **(B)** Kaplan-Meier analysis of overall survival time of EC patients with *PTEN*^L^*ESR*1^L^*PGR*^L^, *PTEN*^L^*ESR1*^H^*PGR*^H^, *PTEN*^H^*ESR1*^L^*PGR*^L^, *PTEN*^H^*ESR1*^H^*PGR*^H^, *PTEN*^L^*ESR1*^L^*PGR*^H^, *PTEN*^L^*ESR1*^H^*PGR*^L^, *PTEN*^H^*ESR1*^H^*PGR*^L^, and *PTEN*^H^*ESR1*^L^*PGR*^H^ phenotypes based on TCGA database. H: high, L: low.

Subsequently, we analyzed the relationship between patients with different EC phenotypes and prognosis, and found that EC patients with PTEN^H^ER^H^PR^H^ phenotype showed a favorable overall survival (OS) time, compared to patients with PTEN^L^ER^L^PR^L^ and PTEN^H^ER^L^PR^L^ phenotype (Fig. 4A). Similarly, EC patients with *PTEN*^H^*ESR1*^H^*PGR*^H^, *PTEN*^L^*ESR1*^H^*PGR*^H^, and *PTEN*^H^*ESR1*^L^*PGR*^H^ phenotypes showed a favorable OS time than patients with *PTEN*^L^*ESR1*^L^*PGR*^L^, and *PTEN*^H^*ESR1*^L^*PGR*^L^ phenotype (Fig. 4B). These studies suggest that triple-high expression of PTEN, ER and PR may predict favorable prognosis in EC patients.

## Discussion

EC is a type of female reproductive malignant tumor, the incidence of which is generally 20~30%. Exploring effective molecular prognosis targets for EC patients has become a hot topic in current research. In 1991, Raju KS, et al. found that post medroxyprogesterone acetate (MPA) dehydrogenase enzyme levels predicted survival more accurately than pre MPA receptor status of the tumors in EC patients 38. Then, some researchers found lack of bcl-2 39, 40, PTEN 41, TIMP-2 42, ER and PR 43, decrease of hemoglobin 44, EpCAM 45, up-regulation of serum CA125 46, serum TIMP-1 47, ulex europeus agglutinin-I (UEA-I) 48, HIF-1α 49, aurora B 50, MMP-7 51, homeobox (HOX) transcript antisense intergenic RNA (HOTAIR) 52, CHRM3 53, AAA+ (ATPases associated with various cellular activities) nuclear coregulator cancer-associated (ANCCA) 54, glycogen synthase kinase-3β (GSK-3β) 55 can serve as a poor prognostic marker for EC patients. Herein, we also found that decrease of *PTEN*,* PGR* and* ESR1* mRNA expression were associated with poor prognosis. However, it was not accurate during follow-up. Especially, we found that some EC patients with decrease of PTEN, but up-regulation of ER or PR, and some EC patients with decrease of ER or PR, but up-regulation of PTEN also have a poor prognosis (data not shown).

These characteristics can also be observed when analyzing *PTEN*,* PGR* and* ESR1* mRNA expression using the TCGA database. Indeed, we found that EC patients can be divided into different phenotypes based on PTEN, ER and PR expression. EC patients were divided into *PTEN*^L^*ESR*1^L^*PGR*^L^, *PTEN*^L^*ESR1*^H^*PGR*^H^, *PTEN*^H^*ESR1*^L^*PGR*^L^, *PTEN*^H^*ESR1*^H^*PGR*^H^, *PTEN*^L^*ESR1*^L^*PGR*^H^, *PTEN*^L^*ESR1*^H^*PGR*^L^, *PTEN*^H^*ESR1*^H^*PGR*^L^, and *PTEN*^H^*ESR1*^L^*PGR*^H^ phenotypes basing on TCGA-UCEC datasets. Similarly, EC tissues can be classified to PTEN^L^ER^L^PR^L^, PTEN^H^ER^L^PR^L^, PTEN^H^ER^H^PR^H^, PTEN^L^ER^H^PR^H^, PTEN^H^ER^H^PR^L^, PTEN^H^ER^L^PR^H^, and PTEN^L^ER^H^PR^L^ phenotypes basing on IHC analysis.

PTEN is a tumor suppressor gene with double phosphatase activity discovered in 1997. It is a homologous gene of phosphatase and tensin which often associated with the deletion of chromosome allele 10q site 23. PTEN plays an important role in the inhibition of tumorigenesis by regulating PTEN/PI3K/AKT, PTEN/FAK/P130cas, PTEN/ERK, p53/MDM2, and FRAP/mTOR signaling pathways. These signal pathways through its lipid phosphatase and protein phosphatase activity, induce apoptosis, block cell cycle, to inhibit tumor cell invasion and metastasis and tumor angiogenesis 13, 14. At present, abnormal expression of PTEN in EC patients has been confirmed by most researchers. In the previous study, we also found that the loss rate of PTEN in EC patients was 58.8% (60/102), and the loss expression of PTEN was closely related to the grade of histological in EC patients, suggesting that the absent expression of PTEN plays an important role on EC occurrence and development 56, 57. Additionally, the expression of ER and PR may be related to the increase of malignancy, the increase of invasion and the transformation of non-hormone-dependent tumors in EC patients 10-16. Therefore, the detection of ER and PR may have important value for EC patients' prognosis and treatment. In particular, it is of great significance to detect ER, PR as endocrine therapy marker in EC patients. To reveal the prognosis of EC patients with different phenotypes based on PTEN, ER and PR expression, 120 cases formalin-fixed paraffin-embedded EC tissues and 543 cases UCEC patients from TCGA-UCEC datasets were analyzed. Results showed that EC patients with triple-high expression of PTEN, ER and PR showed a lower expression of Ki-67, p53 and CA125. Furthermore, triple-high expression of PTEN, ER and PR may predict favorable prognosis in EC patients.

Fiorillo, et al. found that the ER-α mutation Y537S, which associated with the over-expression of a number of protein markers of poor clinical outcome (COL6A3, ERBB2, STAT3, AFP, TFF1, CDK4 and CD44) can confer tamoxifen-resistance via enhanced mitochondrial metabolism, glycolysis and Rho-GDI/PTEN signaling 58. Moreover, PTEN insufficiency stimulated ER^+^ breast cancer cell growth 59. Additionally, PI3K/PTEN/AKT pathway was crucial for many aspects of cell growth and survival, feedback regulation on PTEN/AKT pathway by the ER stress kinase PERK mediated by interaction with the Vault complex^60^. ER-activating ability of breast cancer stromal fibroblasts was regulated independently of alteration of PTEN 61. Herein, basing on IHC analysis, EC tissues can be classified to PTEN^L^ER^L^PR^L^, PTEN^H^ER^L^PR^L^, PTEN^H^ER^H^PR^H^, PTEN^L^ER^H^PR^H^, PTEN^H^ER^H^PR^L^, PTEN^H^ER^L^PR^H^, and PTEN^L^ER^H^PR^L^ phenotypes. And, patients with PTEN^L^ER^L^PR^L^ showed high malignancy, while patients with PTEN^H^ER^H^PR^H^ showed low malignancy. Therefore, combined detection of PTEN, ER, PR may help identify a small subset of EC with more aggressive behavior and may aid in risk stratification.

In summary, our findings indicate that EC patients with triple-high expression of PTEN, ER and PR showed a lower degree of malignancy and proliferative activity and predicted a favorable prognosis. However, the physiological and pathological features of EC patients with different phenotypes basing on PTEN, ER and PR expression still require further investigation.

## Figures and Tables

**Table 1:** The demographic and clinical characteristics for all EC phenotypes

Group	EC phenotypes [n(%)]						
	PTEN^low^ER^low^PR^low^ (n = 48)	PTEN^high^ER^low^PR^low^ (n = 30)	PTEN^high^ER^high^PR^high^ (n = 20)	PTEN^low^ER^high^PR^high^ (n = 12)	PTEN^high^ER^high^PR^low^ (n = 4)	PTEN^high^ER^low^PR^high^ (n = 4)	PTEN^low^ER^high^PR^low^ (n = 2)
**Histological grade**							
G1	10(20.83)	18(60.00)	12(60.00)	5(41.67)	1(25.00)	1(25.00)	1(50.00)
G2	20(41.67)	6(20.00)	5(25.00)	5(41.67)	1(25.00)	1(25.00)	1(50.00)
G3	18(37.50)	6(20.00)	3(15.00)	2(16.67)	2(50.00)	2(50.00)	0(-)
**FIGO clinical stage**							
I	12(25.00)	20(66.67)	9(45.00)	5(41.67)	1(25.00)	2(50.00)	0(-)
II	18(37.50)	4(13.33)	7(35.00)	3(25.00)	1(25.00)	1(25.00)	1(50.00)
III	13(27.08)	4(13.33)	3(15.00)	3(25.00)	1(25.00)	1(25.00)	1(50.00)
IV	5(10.42)	2(6.67)	1(5.00)	1(8.33)	1(25.00)	0(-)	0(-)
**Myometrial tumor invasion**							
Yes	22(45.83)	15(50.00)	8(40.00)	7(58.33)	1(25.00)	2(50.00)	0(-)
No	26(54.17)	15(50.00)	12(60.00)	5(41.67)	3(75.00)	2(50.00)	2(50.00)
Age (years)							
> 45y	30(62.50)	17(56.67)	12(60.00)	7(58.33)	2(50.00)	3(75.00)	1(50.00)
≤ 45y	18(37.50)	13(43.33)	8(40.00)	5(41.67)	2(50.00)	1(25.00)	1(50.00)

## Abbreviations

PTEN: phosphatase and tensin homolog
ER: estrogen receptor
PR: progesterone receptor
EC: Endometrial carcinoma
UCEC: uterine corpus endometrial carcinoma
TCGA: the cancer genome atlas
BMI: Body Mass Index
OS: Overall survival
IHC: Immunohistochemistry
GSK-3β: glycogen synthase kinase-3β
ANT: adjacent normal tissues
